# The Brain Mechanisms Underlying the Perception of Pungent Taste of Capsaicin and the Subsequent Autonomic Responses

**DOI:** 10.3389/fnhum.2015.00720

**Published:** 2016-01-19

**Authors:** Shinpei Kawakami, Hajime Sato, Akihiro T. Sasaki, Hiroki C. Tanabe, Yumiko Yoshida, Mitsuru Saito, Hiroki Toyoda, Norihiro Sadato, Youngnam Kang

**Affiliations:** ^1^Department of Neuroscience and Oral Physiology, Graduate School of Dentistry, Osaka UniversitySuita, Japan; ^2^Morinaga & Co., Ltd., YokohamaJapan; ^3^Division of Cerebral Integration, National Institute for Physiological SciencesOkazaki, Japan; ^4^Pathophysiological and Health Science Team, RIKEN Center for Life Science TechnologiesKobe, Japan; ^5^Department of Physiology, Graduate School of Medicine, Osaka City UniversityOsaka, Japan; ^6^Department of Psychology, Graduate School of Environmental Studies, Nagoya UniversityNagoya, Japan; ^7^Department of Oral Physiology, Graduate School of Medical and Dental Sciences, Kagoshima UniversityKagoshima, Japan

**Keywords:** fMRI, insular cortex, capsaicin, taste recognition, autonomic function

## Abstract

In a human fMRI study, it has been demonstrated that tasting and ingesting capsaicin activate the ventral part of the middle and posterior short gyri (M/PSG) of the insula which is known as the primary gustatory area, suggesting that capsaicin is recognized as a taste. Tasting and digesting spicy foods containing capsaicin induce various physiological responses such as perspiration from face, salivation, and facilitation of cardiovascular activity, which are thought to be caused through viscero-visceral autonomic reflexes. However, this does not necessarily exclude the possibility of the involvement of higher-order sensory-motor integration between the M/PSG and anterior short gyrus (ASG) known as the autonomic region of the insula. To reveal a possible functional coordination between the M/PSG and ASG, we here addressed whether capsaicin increases neural activity in the ASG as well as the M/PSG using fMRI and a custom-made taste delivery system. Twenty subjects participated in this study, and three tastant solutions: capsaicin, NaCl, and artificial saliva (AS) were used. Group analyses with the regions activated by capsaicin revealed significant activations in the bilateral ASG and M/PSG. The fMRI blood oxygenation level-dependent (BOLD) signals in response to capsaicin stimulation were significantly higher in ASG than in M/PSG regardless of the side. Concomitantly, capsaicin increased the fingertip temperature significantly. Although there was no significant correlation between the fingertip temperatures and BOLD signals in the ASG or M/PSG when the contrast [Capsaicin–AS] or [Capsaicin–NaCl] was computed, a significant correlation was found in the bilateral ASG when the contrast [2 × Capsaicin–NaCl–AS] was computed. In contrast, there was a significant correlation in the hypothalamus regardless of the contrasts. Furthermore, there was a significant correlation between M/PSG and ASG. These results indicate that capsaicin increases neural activity in the ASG as well as the M/PSG, suggesting that the neural coordination between the two cortical areas may be involved in autonomic responses to tasting spicy foods as reflected in fingertip temperature increases.

## Introduction

Capsaicin is the pungent ingredient of hot red pepper and has long been traditionally used as ingredient of spices, preservatives, and medicine (Suzuki and Iwai, 1984). In response to tasting and digesting spicy foods containing capsaicin, various physiological responses such as perspiration from face (Lee, 1954), salivation (Dunér-Engström et al., 1986) and increases of systolic blood pressure, heart rate, body core, and surface temperatures (Hachiya et al., 2007) are transiently induced. Such autonomic responses may be induced through viscero-visceral autonomic reflexes (Ganong, 2003). The exact neuroanatomical basis of these reflexes is not firmly established, but it is generally believed that capsaicin activates nociceptive afferents innervating the oral organs and gut, which in turn activates sympathetic nervous system, causing facilitation of cardiovascular activity as a result of viscero-visceral reflex. It is also reported that capsaicin accelerates adrenaline secretion by activating the adrenal sympathetic efferent nerve in rats (Hachiya et al., 2007). However, this does not necessarily exclude the possibility of the involvement of higher-order sensory-motor integration.

Since capsaicin activates the transient receptor potential vanilloid 1 (TRPV1) on primary afferent neurons (Holzer, 1991), capsaicin-induced autonomic reflexes might be caused by impulse activity in nociceptive afferents innervating oral mucosa and taste bud expressing TRPV1 (Ishida et al., 2002; Kido et al., 2003; Sasaki et al., 2013). In the rat insular cortex, the dysgranular region is involved in taste perception as the primary gustatory area (Yamamoto, 1987; Accolla et al., 2007), while its caudal granular region is potentially involved in visceral sensory-motor control as the primary autonomic area (Ruggiero et al., 1987; Cechetto and Saper, 1990; Yasui et al., 1991). Using voltage-sensitive dye imaging and whole cell recording in rat slice preparations, we recently demonstrated that theta-band oscillatory neural coordination between the gustatory and autonomic insular cortices can be induced by activation of TRPV1 in the insular cortex (Saito et al., 2012). Therefore, it may be possible that not only the viscero-visceral reflex but also such a neural coordination induced by TRPV1 activation is responsible for the autonomic responses to tasting and ingesting spicy foods.

Immunohistochemical studies revealed that TRPV1 is expressed in epithelial cells (Marincsák et al., 2009) and taste buds mainly in the circumvallate papillae (Tachibana and Chiba, 2006) of the human tongue. A functional magnetic resonance imaging (fMRI) study in human subjects demonstrated that the tasting and swallowing of 44 μM capsaicin cause excitation in the primary gustatory area, the ventral part of the middle and posterior short gyri (M/PSG) of the insular cortex (Rudenga et al., 2010), suggesting that capsaicin is perceived as hot and spicy tastes. On the other hand, the anterior short gyrus (ASG) of the insular cortex has been identified as the center for the autonomic sensory-motor integration in recent fMRI studies (Craig, 2002; Beissner et al., 2013; Cechetto, 2014). This anatomical arrangement of the gustatory and autonomic areas in the insular cortex is very similar to that of the rat, suggesting a possibility of neural coordination between M/PSG and ASG. However, it has not been investigated whether the oral administration of capsaicin at a higher concentration activates not only M/PSG but also ASG and whether such ASG activation is involved in autonomic responses in human subjects. We here demonstrate that the tasting and ingestion of 65 μM capsaicin activated ASG as well as M/PSG and a significant correlation was found between the effects size of fMRI BOLD signals in the bilateral ASG but not in M/PSG and the fingertip temperature increases.

## Materials and Methods

### Subjects

Experiments were performed on 20 healthy subjects (16 males and four females; aged 20–36 years) without any history of neuromuscular disorder or injury to their brain. Written informed consent was obtained from all subjects before the experiment. Ethical approval from the ethical committee of the National Institute for Physiological Sciences and the ethical committee of Osaka University were obtained before the experiment.

### Stimulus Solution

The following three solutions were used as tastants: artificial saliva (AS), 65 μM capsaicin, and 0.75 M NaCl dissolved in deionized water. The concentrations of capsaicin and NaCl were determined to be approximately equally intense based on psychophysiological tests (Rudenga et al., 2010). NaCl solution at this concentration is widely used as a salty tastant (Spetter et al., 2010; Mascioli et al., 2015), and a limited application of 1 M NaCl did not cause aversive sensation (Mascioli et al., 2015). It was confirmed in all the subjects participated that 0.3 mL NaCl solution at 0.75 M did not cause aversive sensation. AS was composed of a 12.5 mM KCl and 1.25 mM NaHCO_3_ solution similar to the ionic components of saliva (O’Doherty et al., 2001). All solutions were delivered at a room temperature (22–24°C) as any effects of temperature, which is known to be represented in the insular cortex (Craig et al., 2000), could not have contributed to any of the effects described in our investigation.

### Stimulus Delivery System

**FIGURE 1:**
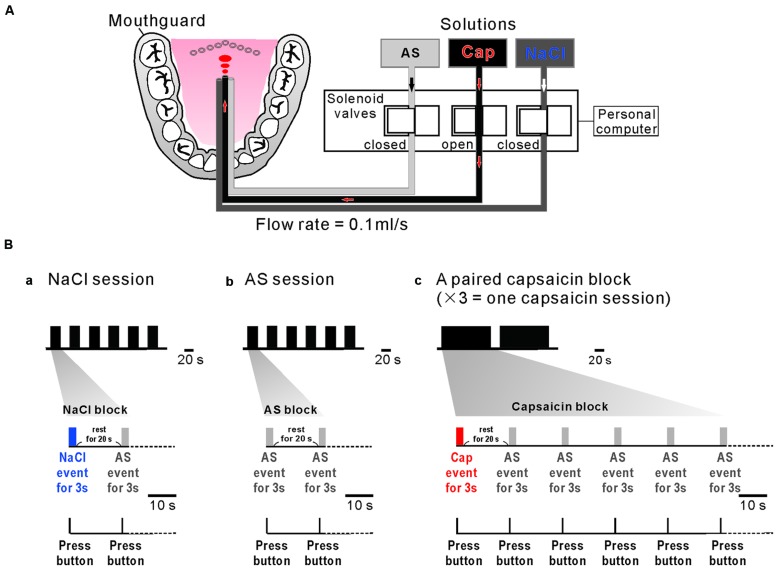
**A custom designed taste delivery system and experimental design. (A)** A schematic diagram of tastants delivery system. Tastants delivered through the three tygon tubes, the tastant flow through which was regulated by the respective solenoid valves that are controlled by a personal computer. Three solutions; artificial saliva (AS), capsaicin (Cap), and NaCl were administered at 0.1 ml/s constant flow rate. **(Ba–c)** NaCl event for 3 s (blue bar) followed by AS event for 3 s (gray bar) with an interevent interval of 20 s was applied six times every 20 s (one NaCl session) **(a)**. AS event for 3 s (gray bar) followed by AS event for 3 s (gray bar) with an interevent interval of 20 s was applied six times every 20 s (one AS session) **(b)**. Capsaicin event (red bar) for 3 s followed by five 3 s AS events (gray bar) applied every 20 s was repeated two times with an interval of 138 s. A paired capsaicin block **(c)** which contains two capsaicin events was repeated 3 times every 10 min (one capsaicin session). Subjects pressed the button as soon as they felt a liquid on their tongue (bottom).

A custom designed taste delivery system was built to administer the liquid stimuli. The three tastants were delivered into the subject’s mouth through the three tygon tubes, one ends of which were connected to the three storage bottles suspended from the ceiling and the other ends were introduced into the mouth to reach the posterior one third of the tongue after the tubes were attached to the incisor region of a rigid custom made mandibular mouthguard with dental resin bonding. The flow of the tastants was controlled by the respective solenoid valves (**Figure 1A**). The opening and shunting of respective solenoid valves, which were placed outside the MRI scanner room, were independently controlled by a personal computer to apply tastant solutions at a constant flow rate of 0.1 ml/s. Tastants were applied to the posterior one third of the subject’s tongue based on the following three rationales: (1) Taste cells which express TRPV1 receptors are mostly located in the circumvallate papillae which are localized in the posterior one third of the human tongue (Tachibana and Chiba, 2006). (2) The strength of compound sensation of taste and burning pain evoked by capsaicin application to the posterior tongue is higher compared to the anterior tongue, regardless of concentrations of capsaicin (Rentmeister-Bryant and Green, 1997; Green and Schullery, 2003). (3) None of taste neurons in the nucleus tractus solitarius in rats displayed prominent excitatory responses to capsaicin application to the anterior tongue (Simons et al., 2003).

### Experimental Design

One NaCl block consisted of a 3 s NaCl event and a 3 s AS event applied after a rest period of 20 s (**Figure 1Ba**), which was repeatedly applied six times every 20 s (one NaCl-session). One AS block consisted of two 3 s AS events separated by 20 s (**Figure 1Bb**), which was repeatedly applied six times every 20 s (one AS-session). A pair of capsaicin blocks, each of which contains a 3 s capsaicin event followed by five 3 s AS events repeated every 20 s, was applied with an inter-block interval of 20 s, consequently spanning about 5 min (**Figure 1Bc**). Because one MRI scan time was limitted to be about 5 min to keep the subject’s attention on the task, a pair of capsaicin blocks was repeated three times every 10 min to constitute one capsaicin session. AS events after taste event application were considered as rinse events. In order to obtain a sufficient number of trials for averaging, each subjects participated in one NaCl-session, one AS-session and one capsaicin-session in the morning and in the same sessions in the afternoon on the same day. Consequently, 24 AS events, 12 NaCl events, and 12 capsaicin events were used for fMRI data analysis.

### Subject Preparation

Subjects were instructed not to change their head positions, and to keep their eyes open to watch a fixation point in front of them. Subjects held a response button in their right hand and pressed the button as soon as possible they felt a liquid touch on their tongue (**Figure 1B**, bottom). The button press was required to average the respective fMRI responses to a tastant which was repeatedly applied, and was used to obtain a positive control for fMRI. And also, they were asked to briefly take a swallow of solution for 1 s between fMRI data scanning so that BOLD signal was not contaminated by movement artifacts related to swallowing. Prior to MRI scan, subjects were given a description of the paradigm and were asked to participate in a training session in a laboratory. The training session served to screen subjects, familiarize subjects with the procedures and equipment used during the actual scan, and make sure that they could press the button at a probability of 90% or higher and take a swallow of solution in correct timing.

### MRI Data Acquisition

All images were acquired using a 3T MR scanner (Allegra; Siemens, Erlangen, Germany). For functional imaging during the sessions, a T2*-weighted gradient-echo echo-planar imaging (EPI) procedure was used to produce 3-mm-thick slices (34 in total) with a 17% gap covering the entire cerebral and cerebellar cortices [repetition time (TR) = 3000 ms; echo time (TE) = 30 ms; flip angle (FA) = 83°; the field of view (FOV) = 192 mm; 64 × 64 matrix with a pixel dimension of 3.0 × 3.0 mm]. The acquisition time (TA) was set at 2000 ms, so as to obtain a 1000-ms “silent period” without any magnetic-field gradient or radiofrequency pulse. This was intended to avoid contaminating motion artifacts by swallowing to the BOLD signal. In total, 960 volumes (96 volumes per run) were acquired. For anatomical imaging, high-resolution whole-brain MR images were also obtained using a T1-weighted three-dimensional (3D) magnetization-prepared rapid-acquisition gradient-echo (MPRAGE) sequence (TR = 2500 ms; TE = 4.38 ms; FA = 8°; FOV = 230 mm; one slab; 192 slices per slab; voxel dimensions = 0.9 mm × 0.9 mm × 1.0 mm).

### Fingertip Temperature Measurement

Body surface temperature at the left little fingertip was measured as indicator of thermogenesis with an electronic thermometer system: thermistor (TSD202A, BIOPAC, Biopac Systems Inc., Goleta, CA, USA), skin temperature amplifier module (SKT100C, BIOPAC, Biopac Systems), data acquisition system (Powerlab, ADInstruments, Colorado Springs, CO, USA) and Power Lab Chart Ver.5 (Powerlab, ADInstruments), during fMRI data acquisition. The TSD202A thermistor which can be reliably used under the condition of 3T-MR scanner (MR conditional) was attached to the skin with surgical tape. Temperature changes following application of tastants were obtained by calculating the fingertip average temperature for 15 s before and after respective sessions as controls and effects of tastants, respectively. The fingertip temperature depends on the rate of blood flow or vascular activity that is regulated by autonomic nervous system (Nilsson, 1987; Allen et al., 2002; Akata et al., 2004; Dhindsa et al., 2008; Tansey et al., 2014; Leung, 2015).

### fMRI Data Processing

The first two volumes of each run were discarded due to unsteady magnetization, and the remaining 94 volumes per run (a total of 940 volumes per subject for 10 runs) were used for the analysis. Image processing and statistical analyses were performed with the Statistical Parametric Mapping package (SPM8; The Wellcome Trust Centre for Neuroimaging, London, UK) implemented in Matlab (Mathworks, Natick, MA, USA). Functional images from each run were realigned to the mean image of all functional images to correct for motion. After the motion correction, the T1-weighted anatomical image was coregistered to the mean image, and then normalized to a standard T1 template image, which defined the Montreal Neurological Institute (MNI) space. The parameters from this normalization process were then applied to each functional image. The spatially normalized EPI images were filtered using a Gaussian kernel of 8 mm full-width at half maximum (FWHM) in the *x*, *y*, and *z* axes.

### fMRI Data Analysis

Initially, we performed a single-subject level analysis. The individual task-related activity was estimated using a general linear model (Friston, 2007). The signal time-course of each subject was modeled with a boxcar function convolved with a canonical haemodynamic-response function (included in SPM8), a high pass filter (with a cut-off period of 128 s), and session effects. For each NaCl run, we included one regressor for NaCl event, one for wash event, and six regressors for six parameters (three displacements and three rotations) from rigid-body realignment stage. For each capsaicin run, we included each one regressor for capsaicin event, one for the first- to fourth-wash event, one for fifth-wash event, and six regressors from the realignment. For the AS run, we included one regressor for AS event and six regressors from the realignment. Serial autocorrelation of the fMRI time series was modeled using a first-order autoregressive model. The resulting set of voxel values for each comparison constituted a statistical parametric map of the *t* statistic [SPM {*t*}].

The weighted sum of the parameters estimated in the individual analyses consisted of “contrast” images, which were used for the group-level analyses. The contrast images obtained from each individual analysis represented the normalized increment of the fMRI signal for each subject. The contrast images of each condition were entered into a flexible factorial model for a multi-subject repeated measured analysis of variance (ANOVA) with subject (one-level for each subject) and event (three levels consisted of capsaicin, NaCl, and AS conditions) factors. To identify regions of overlapping responses to the three or two different tastes events, we performed conjunction analyses with a conjunction null hypothesis. This statistic identifies voxels that are significantly activated in each of the individual contrasts included in the conjunction (Friston et al., 2005). Furthermore, the three types of the contrast of interest [Capsaicin–AS], [Capsaicin–NaCl], and [2 × Capsaicin–NaCl–AS] were computed to reveal the regions that are selectively or more potentially activated by capsaicin. The statistical threshold was set at *p* < 0.05 with correction of the family-wise error (FWE) at the voxel level, and the resulting set of voxel values for each comparison constituted a statistical parametric map of the *t* statistic [SPM{*t*}].

We performed ROI analysis using anatomically defined insular cortex, which was determined by using WFU pickatlas tool (Maldjian et al., 2003). We extracted effect size regarding to each condition from the anatomically defined insula. Numerical data were expressed as the mean ± SD. Then, we assessed statistical significance in non-pairwise and pairwise experiments using repeated-measures ANOVA with Fisher’s protected least significant difference *post hoc* test (STATISTICA 10J, StatSoft), and Pearson’s correlation coefficients between the effect sizes and the fingertip temperature changes. Statistical analysis of the fingertip temperature changes was performed with a paired *t*-test (*p* < 0.05).

## Results

### Conjunction Analysis of All Taste Stimuli

**FIGURE 2:**
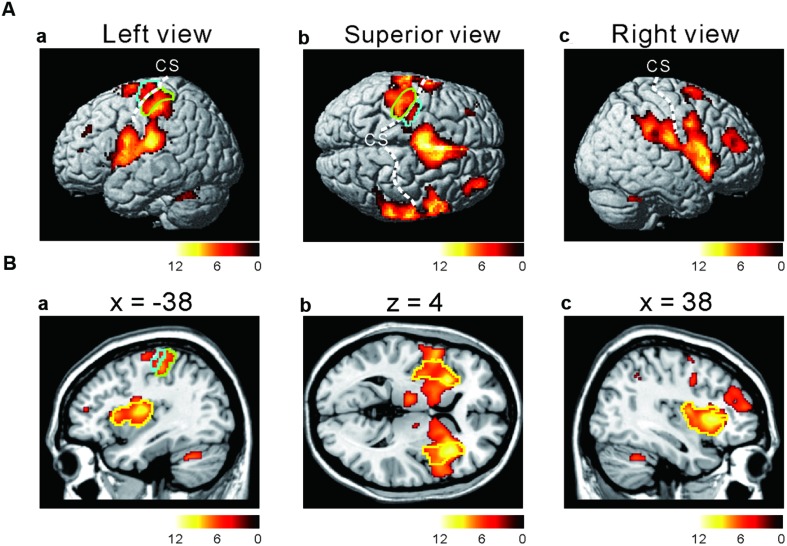
**Active brain regions revealed by a conjunction analysis. (Aa–c)** Lateral **(a,c)** and superior **(b)** views after performing conjunction analyses of three different tastes responses [Capsaicin & NaCl & AS]. Green enclosed areas, postcentral gyrus (S1); Blue enclosed area, precentral gyrus (M1); CS, central sulcus. The color bar represents the *T*-values. **(Ba–c)** Sagittal **(a,c)** and horizontal **(b)** views after performing conjunction analyses of three different tastes responses [Capsaicin & NaCl & AS]. Green enclosed areas, postcentral gyrus (S1); Blue enclosed area, precentral gyrus (M1); yellow enclosed area, insula; CS, central sulcus. The color bar represents the *T*-values.

To investigate which areas are commonly activated by the three taste stimuli, we first performed a conjunction analysis between all the responses to the respective taste stimuli (**Figure 2** and **Table 1**). Most prominently activated brain areas were bilateral anterior insula (-32, 18, 8; *T* = 12.15 and 36, 22, 4; *T* = 10.84), which were included in the largest cluster together with bilateral middle insula (-34, -6, 14; *T* = 9.85 and 38, -2, 12; *T* = 6.61, **Figure 2B**), left postcentral gyrus (-54, -22, 20; *T* = 11.00, **Figures 2Aa,Ba**) and left precentral gyrus (-52, 4, 10; *T* = 9.06, **Figures 2Aa,Ba**) as represented with multiple peaks. These results indicate that the insula and postcentral gyri were activated following application of tastants on subject’s tongue and the precentral gyrus was activated during pressing the button, suggesting that the fMRI data revealing the activation of the insular cortices are reliable. Bilateral supplementary motor area, bilateral middle cingulate cortex, bilateral middle frontal gyrus, and bilateral cerebellum were also activated (**Table 1**). These results were completely the same as the results obtained by performing a conjunction analysis of two different tastes [Capsaicin and NaCl].

**Table 1:** Conjunction analysis on brain regions activated by application of the three different tastants.

MNI coordinates			*T*-value	*Z*-score	Peak *p* (FWE-cor)	Cluster size (mm^3^)	Side	Anatomical labels	Brodmann area
*x*	*y*	*z*							
–32	18	8	12.15	Inf	0.0000	105472	L	Anterior Insula	
–54	–22	20	11.00	8.03	0.0000		L	Postcentral Gyrus	41
36	22	4	10.84	7.96	0.0000		R	Anterior Insula	13
–4	–2	64	9.78	7.46	0.0000	36928	L	Supplementary Motor area	6
6	12	58	9.69	7.42	0.0000		R	Supplementary Motor area	
–8	4	40	9.59	7.37	0.0000		L	Middle Cingulate Cortex	24
36	50	16	8.41	6.76	0.0000	4352	R	Middle Frontal Gyrus	
42	36	24	6.22	5.41	0.0012		R	Inferior Frontal Gyrus (p. Triangularis)	
34	36	20	5.97	5.24	0.0028		R	Middle Frontal Gyrus	
20	–54	–20	7.59	6.28	0.0000	4488	R	Cerebellum	
38	–52	–34	6.32	5.48	0.0009		R	Cerebellum	
–34	–50	–32	7.43	6.19	0.0000	3752	L	Cerebellum	
–24	–64	–24	6.98	5.91	0.0001		L	Cerebellum	
6	–26	30	6.20	5.40	0.0013	888	R	Middle Cingulate Cortex	
–4	–26	30	5.79	5.11	0.0051		L	Cingulate Gyrus	
–32	38	32	5.92	5.21	0.0033	304	L	Middle Frontal Gyrus	
–10	–76	–30	5.71	5.06	0.0065	544	L	Cerebellum	
–40	46	14	5.41	4.84	0.0169	80	L	Middle Frontal Gyrus	
12	–16	–6	5.33	4.78	0.0222	80	R	Brainstem	
40	0	60	5.29	4.75	0.0249	72	R	Middle Frontal Gyrus	
12	–22	–4	5.20	4.68	0.0328	16	R	Brainstem	
–60	–40	20	5.18	4.67	0.0350	8	L	SuperiorTemporal Gyrus	22

*The statistical threshold size of activation was set at *p* < 0.05 with correction of the family-wise error (FWE) at the voxel level, when the height threshold was set at *T* > 5.06. (*x, y, z*) values represent Montreal Neurological Institute (MNI) coordinates (mm). R, right; L, Left*.

### The Cortical Regions that Display Stronger Responses to Capsaicin Compared to NaCl or AS

**FIGURE 3:**
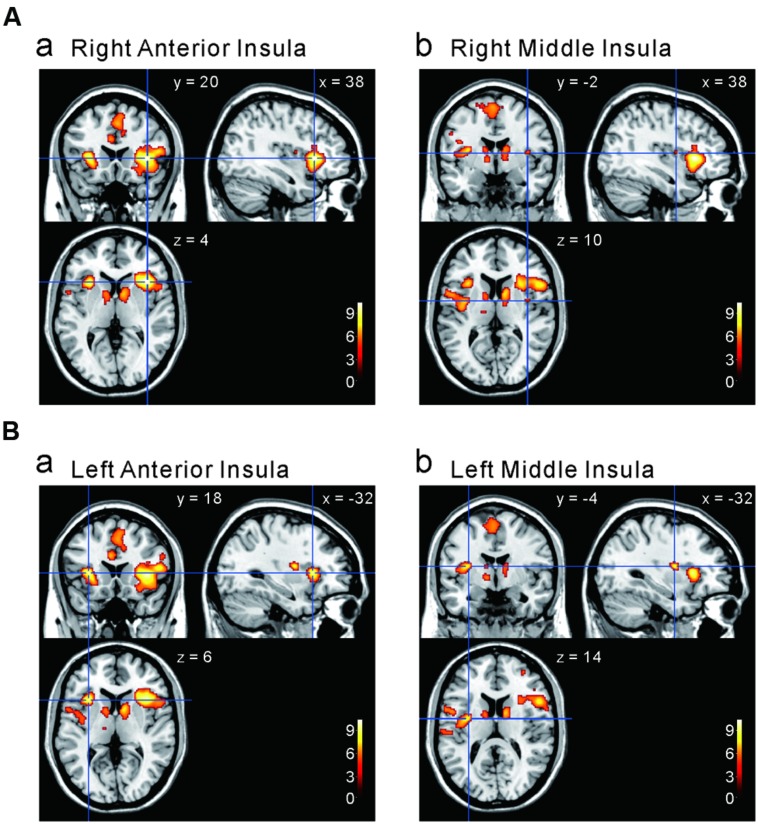
**Brain areas significantly activated by the comparison [Capsaicin–AS]. (A,B)** The anterior and middle insula activated by capsaicin stimuli. (*x, y, z*) values represent Montreal Neurological Institute (MNI) coordinates (mm), and the color bar represents the *T*-values.

**FIGURE 4:**
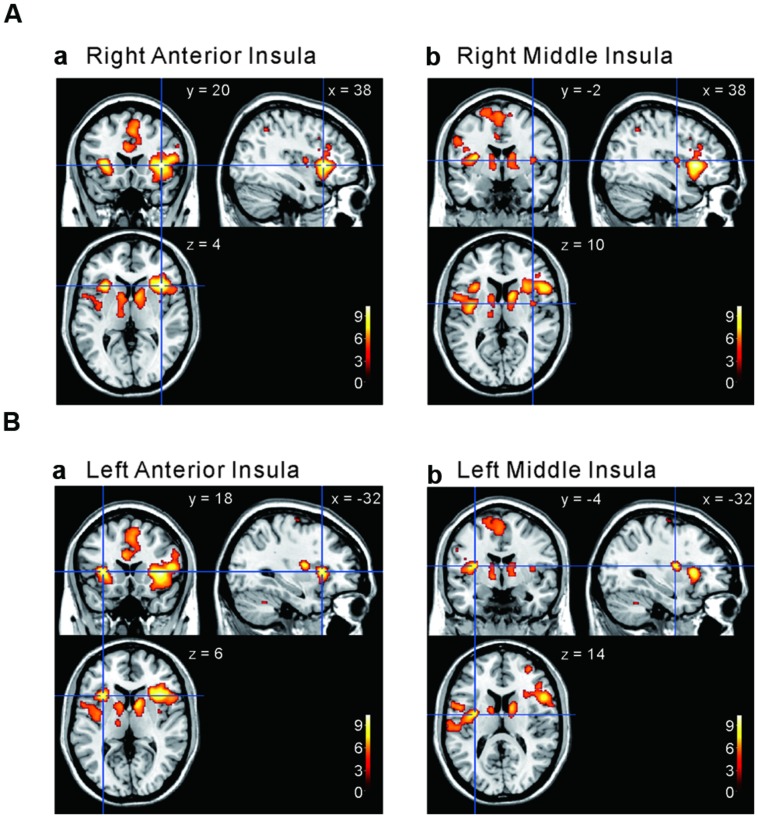
**Brain areas significantly activated by the comparison [Capsaicin–NaCl]. (A,B)** The anterior and middle insula activated by capsaicin stimuli. (*x, y, z*) values represent Montreal Neurological Institute (MNI) coordinates (mm), and the color bar represents the *T*-values.

We next investigated the cortical regions which are more strongly activated by capsaicin than by NaCl or AS. First, we performed the group analyses of the two types of comparisons; [Capsaicin–AS] (**Figure 3** and **Table 2**) and [Capsaicin–NaCl] (**Figure 4** and **Table 3**). Activated brain areas revealed by the two comparisons were the bilateral anterior insula and bilateral middle insula (**Figures 3** and **4**). The MNI coordinates of peak voxels in anterior and middle insula were the same between the two comparisons. However, no brain areas were found to be significantly activated with the comparison [NaCl–AS] (data not shown; see Discussion).

**Table 2:** The peak coordinates displayed significant responses by the comparison [Capsaicin–AS].

MNI coordinates			*T*-value	*Z*-score	Peak *p* (FWE-cor)	Cluster size (mm^3^)	Side	Anatomical labels	Brodmann area
*x*	*y*	*z*							
38	20	4	10.06	7.60	0.0000	15296	R	Anterior Insula	13
52	16	12	8.50	6.80	0.0000		R	Inferior Frontal Gyrus (p. Opercularis)	44
52	8	26	7.71	6.35	0.0000		R	Inferior Frontal Gyrus (p. Opercularis)	9
–32	18	6	9.49	7.32	0.0000	2800	L	Anterior Insula	13
–22	10	–12	5.41	4.84	0.0170		L	Putamen	
6	32	44	8.04	6.55	0.0000	15040	R	Medial Frontal Gyrus	8
10	10	60	7.38	6.16	0.0000		R	Supplementary Motor area	6
–6	16	26	7.00	5.92	0.0001		L	Anterior Cingulate	24
–32	–4	14	7.81	6.41	0.0000	5224	L	Middle Insula	13
–40	–8	12	7.04	5.95	0.0001		L	Rolandic Operculum	13
–52	4	12	6.72	5.74	0.0002		L	Precentral Gyrus	
–54	–22	20	7.75	6.38	0.0000	2968	L	Postcentral Gyrus	40
–62	–20	20	6.96	5.90	0.0001		L	Postcentral Gyrus	40
–60	–26	30	5.93	5.21	0.0032		L	Inferior Parietal Lobule	40
10	6	6	7.37	6.15	0.0000	2208	R	Caudate Nucleus	
64	–18	24	6.64	5.69	0.0003	752	R	Postcentral Gyrus	1
–8	–10	–2	6.35	5.50	0.0008	1872	L	Thalamus (including Hypothalamus)	
–12	6	4	5.96	5.23	0.0029		L	Caudate Nucleus	
–12	0	14	5.85	5.15	0.0042		L	Caudate Nucleus	
34	50	14	5.52	4.92	0.0121	96	R	Middle Frontal Gyrus	10
48	30	22	5.36	4.80	0.0200	104	R	Middle Frontal Gyrus	
38	–2	10	5.31	4.77	0.0233	40	R	Middle Insula	13
–14	–16	10	5.27	4.73	0.0267	48	L	Thalamus	
46	30	14	5.14	4.64	0.0389	8	R	Inferior Frontal Gyrus	46
40	28	26	5.09	4.60	0.0458	8	R	Middle Frontal Gyrus	
–50	–34	48	5.08	4.59	0.0474	8	L	Inferior Parietal Lobule	40

*The statistical threshold size of activation was set at *p* < 0.05 with correction of the family-wise error (FWE) at the voxel level. (*x, y, z*) values represent Montreal Neurological Institute (MNI) coordinates (mm). R, right; L, Left*.

**Table 3:** The peak coordinates displayed significant responses by the comparison [Capsaicin–NaCl].

MNI coordinates			*T*-value	*Z*-score	Peak *p* (FWE-cor)	Cluster size (mm^3^)	Side	Anatomical labels	Brodmann area
*x*	*y*	*z*							
38	20	4	10.38	7.74	0.0000	19016	R	Anterior Insula	13
52	16	12	9.06	7.10	0.0000		R	Inferior Frontal Gyrus (p. Opercularis)	44
52	8	26	8.43	6.76	0.0000		R	Inferior Frontal Gyrus (p. Opercularis)	9
–32	18	6	9.69	7.42	0.0000	3224	L	Anterior Insula	13
–22	10	–10	5.60	4.98	0.0092		L	Putamen	
–32	–4	14	8.55	6.83	0.0000	15312	L	Middle Insula	13
–54	–22	20	8.50	6.80	0.0000		L	Postcentral Gyrus	40
–62	–20	20	7.70	6.35	0.0000		L	Postcentral Gyrus	40
12	8	8	7.94	6.49	0.0000	3432	R	Caudate Nucleus	
20	0	0	5.61	4.98	0.0091		R	Pallidum	
12	–12	0	5.26	4.73	0.0272		R	Thalamus	
4	32	44	7.93	6.48	0.0000	20936	R	Medial Frontal Gyrus	8
10	10	60	7.87	6.45	0.0000		R	Supplementary Motor Area	6
–4	16	26	7.24	6.07	0.0000		L	Anterior Cingulate	24
64	–16	24	7.49	6.23	0.0000	2560	R	Postcentral Gyrus	1
60	–20	44	5.67	5.03	0.0075		R	Precentral Gyrus	2
56	–28	48	5.55	4.94	0.0109		R	Postcentral Gyrus	40
–8	–10	–2	6.68	5.72	0.0003	3400	L	Thalamus (including Hypothalamus)	
–12	6	4	6.35	5.50	0.0008		L		
–12	2	12	6.13	5.35	0.0016		L	Caudate Nucleus	
34	50	14	6.19	5.39	0.0014	272	R	Middle Frontal Gyrus	10
38	–2	10	5.75	5.09	0.0058	232	R	Middle Insula	13
36	0	2	5.21	4.69	0.0319		R	Middle Insula	
34	14	36	5.73	5.07	0.0061	72	R	Middle Frontal Gyrus	9
–32	–12	68	5.45	4.87	0.0148	40	L	Precentral Gyrus	6
–42	–24	56	5.44	4.86	0.0157	160	L	Postcentral Gyrus	3
38	–50	46	5.36	4.80	0.0200	72	R	Inferior Parietal Lobule	40
18	–52	–20	5.32	4.77	0.0229	56	R	Cerebellum	
–32	–52	–32	5.22	4.70	0.0309	40	L	Cerebellum	
20	4	60	5.21	4.69	0.0320	8	R	Superior Frontal Gyrus	
38	14	22	5.07	4.59	0.0476	8	R	Inferior Frontal Gyrus (p. Trianqularis)	
–44	–10	60	5.07	4.59	0.0486	8	L	Precentral Gyrus	6

*The statistical threshold size of activation was set at *p* < 0.05 with correction of the family-wise error (FWE) at the voxel level. (*x, y, z*) values represent Montreal Neurological Institute (MNI) coordinates (mm). R, right; L, Left*.

**FIGURE 5:**
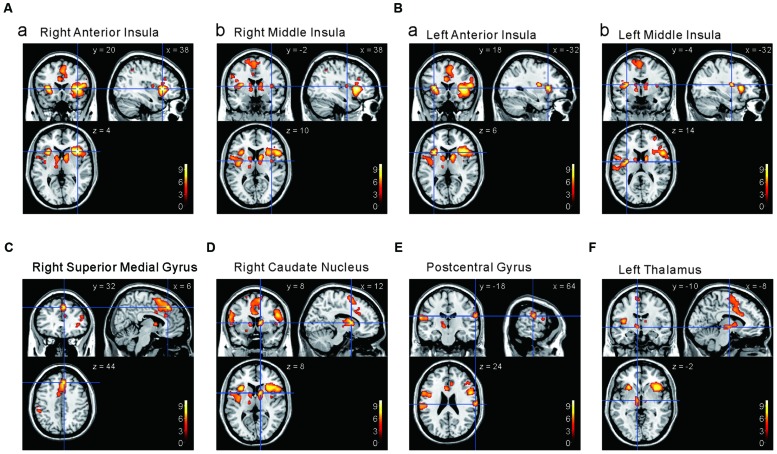
**Brain areas significantly activated by the comparison [2 × Capsaicin–NaCl–AS]. (A,B)** The anterior and middle insula activated by capsaicin stimuli. **(C–F)** Brain areas particularly activated by capsaicin stimuli. (*x, y, z*) values represent Montreal Neurological Institute (MNI) coordinates (mm), and the color bar represents the *T*-values.

We next performed the group analysis of the comparison [2 × Capsaicin–NaCl–AS] to reveal which voxels were significantly and more potentially activated by capsaicin compared to NaCl or AS (**Figure 5** and **Table 4**). Brain areas particularly activated by capsaicin stimuli were the bilateral anterior insula (**Figures 5Aa,Ba**), bilateral middle insula (**Figures 5Ab,Bb**), right superior medial gyrus (**Figure 5C**), right caudate nucleus (**Figure 5D**), postcentral gyrus (**Figure 5E**), ventral posteromedial nucleus (VPM) of left thalamus (**Figure 5F**). Highly significant activations were found in the following coordinates; [38, 20, 4] (*T* = 10.43; the right anterior insula), [–32, 18, 6] (*T* = 9.79; the left anterior insula), [–32, –4, 14] (*T* = 8.36; the left middle insula) and [–6, 16, 26] (*T* = 7.26; the left anterior cingulate cortex). These cortices are known to play crucial roles for blood pressure control, among human central autonomic network (Nagai et al., 2010). Significant activation was also observed in the hypothalamus [–6, –8, –2] (*T* = 5.41), which is the subcortical autonomic control center (Nakamura, 2011). This region was included in a cluster of left thalamus (**Table 4**).

**Table 4:** The peak coordinates displayed significant responses by the comparison [2 × Capsaicin–NaCl–AS].

MNI coordinates			*T*-value	*Z*-score	Peak *p* (FWE-cor)	Cluster size (mm^3^)	Side	Anatomical labels	Brodmann area
*x*	*y*	*z*							
38	20	4	10.43	7.76	0.0000	18600	R	Anterior Insula	13
52	16	12	8.96	7.05	0.0000		R	Inferior Frontal Gyrus (p. Opercularis)	44
52	8	26	8.24	6.66	0.0000		R	Inferior Frontal Gyrus (p. Opercularis)	9
–32	18	6	9.79	7.47	0.0000	3272	L	Anterior Insula	13
–22	10	–12	5.62	4.99	0.0087		L	Subcallosal Gyrus	
–32	–4	14	8.36	6.72	0.0000	12208	L	Middle Insula	13
–54	–22	20	8.30	6.69	0.0000		L	Postcentral Gyrus	40
–40	–8	12	7.50	6.23	0.0000		L	Rolandic Operculum	13
6	32	44	8.12	6.59	0.0000	19832	R	Superior Medial Gyrus	8
10	10	60	7.79	6.40	00000		R	Supplementary Motor area	6
–6	16	26	7.26	6.09	0.0000		L	Anterior Cingulate	24
12	8	8	7.82	6.42	0.0000	18600	R	Caudate Nucleus	
64	–18	24	7.21	6.06	0.0000	1344	R	Postcentral Gyrus	1
–8	–10	–2	6.65	5.70	0.0003	3016	L	Thalamus (including Hypothalamus)	
–12	6	4	6.28	5.45	0.0010		L	Caudate Nucleus	
–12	2	12	6.10	5.33	0.0018		L	Caudate Nucleus	
34	50	14	5.98	5.24	0.0028	240	R	Middle Frontal Gyrus	10
38	–2	10	5.65	5.01	0.0080	168	R	Middle Insula	13
38	–50	46	5.32	4.77	0.0227	48	R	Inferior Parietal Lobule	40
–42	–24	56	5.28	4.75	0.0254	40	L	Postcentral Gyrus	3
–32	–12	68	5.22	4.70	0.0309	16	L	Precentral Gyrus	6
60	–20	44	5.18	4.67	0.0348	40	R	Precentral Gyrus	2
34	14	36	5.11	4.62	0.0425	8	R	Middle Frontal Gyrus	9
–52	–32	56	5.09	4.60	0.0457	16	L	Postcentral Gyrus	40
18	–52	–20	5.09	4.60	0.0460	16	R	Cerebellum	
12	–12	0	5.09	4.60	0.0462	24	R	Thalamus	

*The statistical threshold size of activation was set at *p* < 0.05 with correction of the family-wise error (FWE) at the voxel level. (x, y, z) values represent Montreal Neurological Institute (MNI) coordinates (mm). R, right; L, Left*.

### Differential Activation of the Short Insular Gyri Following Capsaicin Application

**FIGURE 6:**
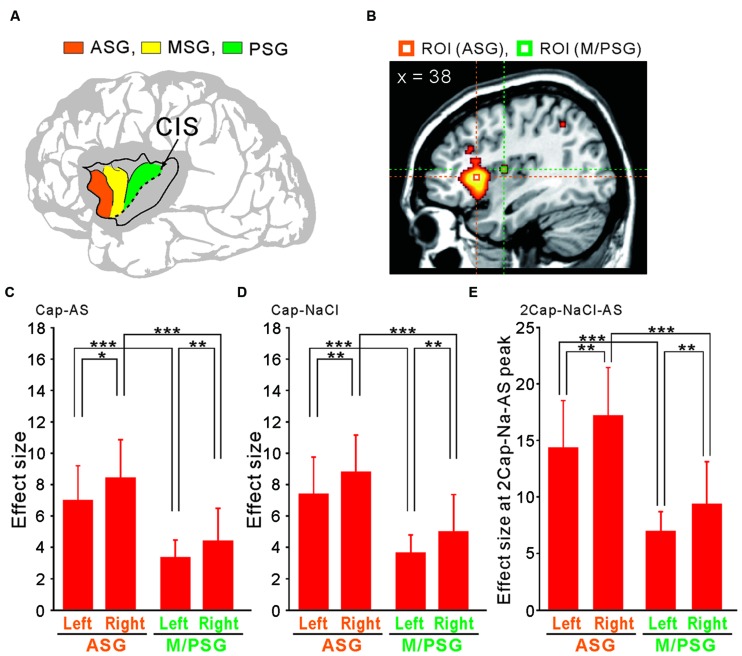
**ROI analysis using anatomically defined coordinates of the insular cortex. (A)** The schema of three anterior short gyri. ASG, the anterior short gyri; MSG, the middle short gyri; PSG, the posterior short gyri. **(B)** Two ROIs located on a right sagittal view, which were at the peak coordinates in the right ASG and M/PSG of the comparison [2 × Capsaicin–NaCl–AS]. **(C–E)** The effect sizes at the bilateral ASG (orange) and M/PSG (green) of the three types of the comparisons; [Capsaicin–AS], [Capsaicin–NaCl], and [2 × Capsaicin–NaCl–AS]. ^∗^*p* < 0.05; ^∗∗^*p* < 0.01; ^∗∗∗^*p* < 0.001.

The human insular cortex usually contains five major gyri: the anterior three gyri (**Figure 6A**) referred to as the “short” gyri and the posterior two gyri termed as the “long” gyri (Türe et al., 1999). The three short gyri were termed as the ASG and M/PSG. To investigate the possible differential activation in the insular cortex, ROI analysis was made at the peak coordinates (**Figure 6B**) in ASG and M/PSG obtained in the group analysis of the three types of the comparisons; [Capsaicin–AS], [Capsaicin–NaCl], and [2 × Capsaicin–NaCl–AS]. Regardless of the types of comparisons, the effect sizes at the ASG were significantly higher than those at the M/PSG (**Figures 6C–E**), suggesting that the ASG was more potentially activated by capsaicin compared to the M/PSG. The effect sizes at the right ASG and M/PSG following capsaicin application were significantly higher compared to the left corresponding gyri (**Figures 6C–E**, compare left and right red bars).

### Correlation Between Fingertip Temperatures and BOLD Signals

**FIGURE 7:**
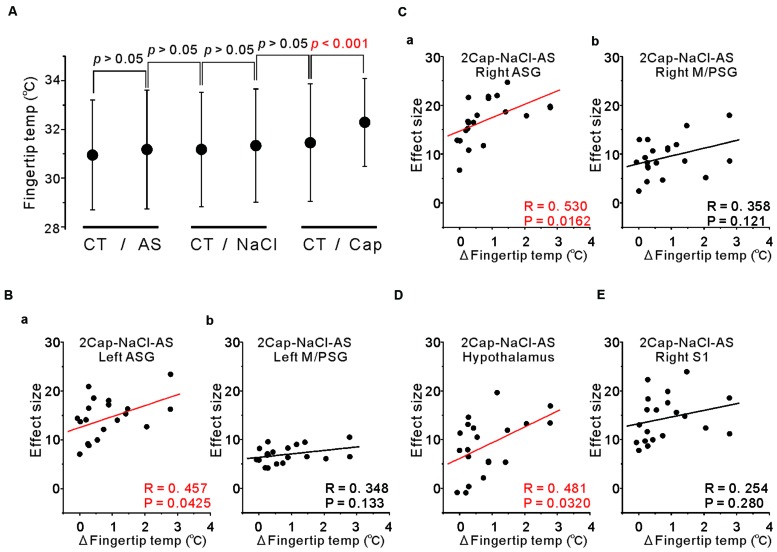
**Correlation between fingertip temperatures and effect sizes at the peak coordinates. (A)** The fingertip temperature changes calculated by averaging for 15 s before and after AS, NaCl and capsaicin sessions. Statistical analyses were performed with a paired *t*-test. CT; control. **(B)** Correlation between the fingertip temperature increases and the effect sizes at respective peak coordinates in the left ASG **(a)** and in the left M/PSG **(b)** obtained in the group analysis of the comparison [2 × Capsaicin–NaCl–AS]. **(C)** Correlation between the fingertip temperature increases and the effect sizes at respective peak coordinates in the right ASG **(a)** and in the right M/PSG **(b)** obtained in the group analysis of the comparison [2 × Capsaicin–NaCl–AS]. **(D)** Correlation between the fingertip temperature increases and the effect sizes at the peak coordinates in the hypothalamus (–6, –8, –2) obtained in the group analysis of the comparison [2 × Capsaicin–NaCl–AS]. **(E)** Correlation between the fingertip temperature increases and the effect sizes at the peak coordinates in the right S1 of postcentral gyrus (64, –18, 24) obtained in the group analysis of the comparison [2 × Capsaicin–NaCl–AS].

To examine the functional relevance of the more increased activity in the ASG and/or M/PSG in response to capsaicin administration compared to NaCl or AS, we measured the fingertip temperatures before and after the respective tastants application. The mean temperature changes following application of NaCl and AS were insignificant and smaller (0.16 ± 0.47 and 0.23 ± 0.74°C, respectively), while the mean temperature increase following application of capsaicin was significant and larger (0.83 ± 0.85°C; **Figure 7A**).

To investigate which areas are more closely involved in the fingertip temperature increases following capsaicin application, we performed correlation analysis between the fingertip temperature increases and the effect sizes of BOLD signals at respective peak coordinates of the three types of the comparisons; [Capsaicin–AS], [Capsaicin–NaCl] and [2 × Capsaicin–NaCl–AS]. There were no significant correlations between the fingertip temperature increases and the effect sizes in the coordinates of both the ASG and M/PSG found as significant in the group analysis of the two comparisons; [Capsaicin–AS] (*p* > 0.05) and [Capsaicin–NaCl] (*p* > 0.05). In contrast, the bilateral ASG in the coordinate found as significant by the comparison [2 × Capsaicin–NaCl–AS] showed a significant positive correlation between fingertip temperatures and its BOLD signals (**Figures 7Ba,Ca**), while the M/PSG did not show any significant correlation regardless of the side (**Figures 7Bb,Cb**). Significant correlations were also found in the bilateral VPM of thalamus, left ventral posterolateral nucleus (VPL) of thalamus, right medial dorsal nucleus (MD) of thalamus (Data not shown) and in the hypothalamus regardless of the comparisons (**Figure 7D**) while no significant correlation was found in the right S1 (**Figure 7E**).

### Coordination Between ASG and M/PSG and Between Diencephalon and ASG or M/PSG.

**FIGURE 8:**
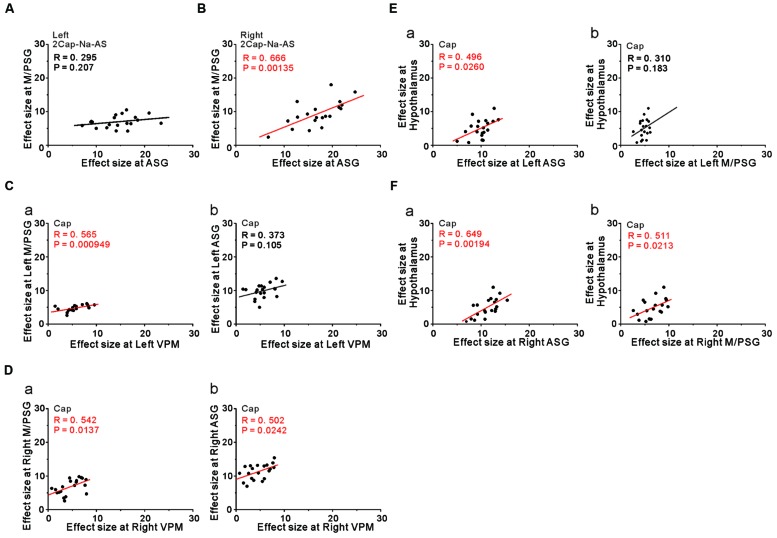
**The integration and coordination between two brain regions. (A,B)** Correlation between the effect sizes at the peak coordinates in the left ASG and left M/PSG **(A)** and those in the right ASG and right M/PSG **(B)** obtained in the group analysis of the comparison [2 × Capsaicin–NaCl–AS]. **(C)** Correlation between the effect sizes at the peak coordinates in the left VPM and left M/PSG **(a)** and those in the left VPM and left ASG **(b)** obtained in the group analysis of the capsaicin responses. **(D)** Correlation between the effect sizes at the peak coordinates in the right VPM and right M/PSG **(a)** and those in the right VPM and right ASG **(b)** obtained in the group analysis of the capsaicin responses. **(E)** Correlation between the effect sizes at the peak coordinates in the left ASG and hypothalamus **(a)** and those in the left M/PSG and hypothalamus **(b)** obtained in the group analysis of the capsaicin responses. **(F)** Correlation between the effect sizes at the peak coordinates in the right ASG and hypothalamus **(a)** and those in the right M/PSG and hypothalamus **(b)** obtained in the group analysis of the capsaicin responses.

We then performed correlation analysis between the effect sizes in the coordinates of the two cortical regions as found significant in the group analysis of the comparison [2 × Capsaicin–NaCl–AS], given the integration and coordination between the two cortical regions. There was a significant positive correlation between the effect sizes of BOLD signals of the right ASG and the right M/PSG (**Figure 8B**), while there was no significant correlation between left ASG and the left M/PSG (**Figure 8A**). Furthermore, no significant correlations were also found between the ASG and M/PSG in the coordinates found as significant in the group analysis of the comparison [Capsaicin–AS] or [Capsaicin–NaCl], (see Discussion). These results suggest the neural coordination between the right M/PSG and ASG potentially in response to capsaicin application as well as the presence of non-linear neural integration among different sensory modalities that occurs during the respective tastants application (see Discussion).

However, to reveal the neural interaction between the subcortical brain region and the cortical region, we performed the correlation analysis between the effect sizes in the coordinates found as significant in the group analysis of the capsaicin responses because there would be no neural integration in the subcortical brain regions. The left and right VPM were significantly correlated with the effect sizes of the left and right M/PSG, respectively (**Figures 8Ca,Da**, respectively), as the solitary tract carrying the primary taste information projects to the M/PSG through the VPM. The left VPM was not significantly correlated with the left ASG (**Figure 8Cb**) while the right VPM was significantly correlated with the right ASG (**Figure 8Db**). These results strongly suggest the neural coordination between the right ASG and M/PSG. Furthermore, there were significant positive correlations between the effect size in the hypothalamus and those in the bilateral ASG (**Figures 8Ea,Fa**) and the right M/PSG (**Figure 8Fb**), but not the left M/PSG (**Figure 8Eb**).

## Discussion

The aim of this study was to investigate whether capsaicin activates the gustatory insular cortex as well as the autonomic insular cortex. We performed the group analyses of the three types of the comparison; [Capsaicin–AS], [Capsaicin–NaCl], and [2 × Capsaicin–NaCl–AS]. Regardless of a difference in these types of comparisons, the significant increases in BOLD signals were observed in the bilateral ASG and M/PSG (**Figures 3–5** and **Tables 2**–**4**), and also the effect sizes in the left and right ASG obtained in the group analyses of three types of the comparisons were significantly higher than those in the left and right M/PSG (**Figures 6C–E**). The fingertip temperature measured after capsaicin application was significantly higher compared to the control whereas no significant temperature changes were observed following application of NaCl or AS (**Figure 7A**). The bilateral ASG in the coordinate found as significant by the comparison [2 × Capsaicin–NaCl–AS] showed a significant positive correlation between its effect sizes and fingertip temperatures (**Figures 7Ba,Ca**). These results suggest that capsaicin activated the ASG more selectively and potentially compared to the M/PSG, which may be responsible for autonomic responses reflected in fingertip temperature increases.

### Can the Tasteless AS be the Control for Tastants in fMRI Responses?

Because AS is tasteless solution, the response to AS has been considered as a control that simply causes tactile sensation in the gustatory insular cortex (de Araujo et al., 2003), and subsequently in many studies (O’Doherty et al., 2001; Frank et al., 2006; Chambers et al., 2009; Nakamura et al., 2011), fMRI response to AS was subtracted from those to other taste stimulations. However, in terms of the intensity of the response and the spatial pattern of the excitation in the gustatory insular cortex, it is questionable whether the AS response can be treated as a control. First, it is well known that many different pyramidal neurons, each of which respond to a different stimulus modality, such as tactile, pressure, cold and warm temperatures, pain, and tastes, are intermingled in the gustatory area and there also exist such neurons that respond to multimodal stimulations (Cechetto and Saper, 1987; Yamamoto et al., 1988; Allen et al., 1991; Hanamori et al., 1998). All these neurons may be synaptically connected, and non-linear summation of synaptic inputs would take place in respective pyramidal neurons in the gustatory insular cortex in response to any taste stimulation. Thus, a taste recognition occurs in the gustatory insular cortex as a result of non-linear integration of many neuronal activities induced by stimulation of various sensory modalities with a tastant. Then, the subtraction of the AS response from some taste response may not necessarily reveal the pure taste response. Second, fMRI studies demonstrated that respective tastes were represented as specific patterns with considerable overlaps in the gustatory cortex (Schoenfeld et al., 2004; Spetter et al., 2010). This suggests that taste recognition is mediated by the activity of a different subset of cell assembly representing differential spatial pattern of excitation, similar to that observed in rats (Accolla et al., 2007) although it was also reported that each taste quality was represented as a discrete hot spot in the gustatory cortex in mice (Chen et al., 2011; Peng et al., 2015). Water also causes a spatial pattern of excitation, which was not the same as the overlapping area of any two of four basic tastants (Accolla et al., 2007). If this is also the case in human subjects, these observations suggest that subtraction of water-like AS response from the response to some tastant may not necessarily reflect the pure taste response and is not the right way of evaluation of taste response. Therefore, provided that AS is a tastant that causes a sensation of tasteless, the group analysis of the comparison [2 × Capsaicin–NaCl–AS] can be an estimate of the area that shows selective or significantly more potential responses to capsaicin application compared to NaCl or AS. The fMRI responses in the left hand area of precentral gyrus (M1) induced by button presses which were performed as soon as the subjects detected the arrival of tastants on the tongue before perceiving tastes did not vary depending on the taste difference among the three tastants, as revealed by the abolishment of the M1 activation by computing the contrast of interest [2 × Capsaicin–NaCl–AS]. Then, even if the button press affects the fMRI taste responses, the button press would cause the same effect on the taste fMRI responses regardless of the different tastants. Therefore, the computing of the contrast of interest [2 × Capsaicin–NaCl–AS] would isolate the taste responses in the taste-associated brain regions to capsaicin application by canceling the possible overlapping activity. Indeed, there were no significant correlations between the temperature changes and the effect sizes in the coordinate found as significant by the comparison [Capsaicin–AS] or [Capsaicin–NaCl] whereas there was a significant correlation between the temperature changes and the effect size in the coordinate found as significant by the comparison [2 × Capsaicin–NaCl–AS].

### Reliability of Fingertip Temperature Measurements During MR Scanning

In this study, capsaicin increased the fingertip temperature significantly. The radio frequency (RF) transmitted from the electromagnetic coil may cause a slight increase in the core body temperature inside the MRI bore. However, the left hand little finger is outside the bore, and the temperature loss will occur during blood flow through the forearm into the peripheral endartery in the little finger. Therefore, it is unlikely that MR scanning causes significant changes in the fingertip temperature of the subjects without application of tastants that activate autonomic nervous system. This is also supported by the observation that the temperature changes observed following application of NaCl or AS were statistically insignificant and much smaller than that observed following application of capsaicin (**Figure 7A**). We calculated the fingertip average temperature for 15 s before and after an entire capsaicin session as a control and an effect of capsaicin application, respectively. In this case, the interval between the two measurements was 35 min (the duration of one capsaicin session, during which a paired capsaicin block repeated three times with 10 min interval), which would be long enough for the development of autonomic responses. Even if the fingertip temperature were increased by RF, 10 min interval is good enough for the recovery of skin temperature to the original value (Adair and Berglund, 1986), in contrast to the cumulative effects of capsaicin.

### Differential Activation Between the ASG and the M/PSG in Response to Capsaicin Application

The M/PSG responds not only to pure taste stimuli as the primary gustatory area but also to stimulation of other intra-oral sensations as an integrated oral sensory region that plays a crucial role in feeding behavior (Small, 2010). It has been demonstrated by an fMRI study in human subjects that tasting and swallowing of capsaicin caused excitation in the M/PSG (Rudenga et al., 2010), suggesting that activation of oral TRPV1 receptors by capsaicin caused the hot and spicy sensation in the primary gustatory area of M/PSG. Partly consistent with this previous study, we found that the oral administration of capsaicin activated the bilateral M/PSG while the bilateral ASG were also activated by capsaicin (**Figures 3A,B**, **4A,B,** and **5A,B**). However, as revealed by the computing of the contrast of interest [2 × Capsaicin–NaCl–AS], the *T*-value and *Z*-score were higher in the ASG than in the M/PSG (**Table 4**). Consistent with this observation, the ROI analysis also revealed that in response to capsaicin administration, the effect sizes in the ASG were significantly larger than those in the M/PSG (**Figures 6C–E**). These observations suggest that capsaicin may have more significantly and strongly activated the ASG compared to the M/PSG.

Regardless of the ASG or the M/PSG, the effect sizes were significantly larger in response to capsaicin application compared to NaCl or AS application. Furthermore, in spite of the taste difference between salty NaCl and tasteless AS, there were no differences in the effect sizes between the ASG and M/PSG in response to AS or NaCl and no differences in the effect sizes between the responses to NaCl and AS in the ASG or in the M/PSG. Consistent with this observation, there were no significant brain areas with the comparison [NaCl–AS] (data not shown). However, it should be noted that tastants were delivered to the posterior part of the tongue which expresses TRPV1 receptors more densely compared to the anterior part and is innervated by the glossopharyngeal nerve (Spector et al., 1990) while salty taste of NaCl is mostly sensed in the anterior part which is solely innervated by the chorda tympani nerve (Oakley, 1967). Therefore, the comparison of effects sizes between capsaicin and NaCl or AS or between NaCl and AS does not necessarily reflect the modality difference in the insular cortices. Nevertheless, it can be at least concluded that capsaicin activated the ASG more selectively and potentially compared to the M/PSG.

### Autonomic Insular Cortex Activated by Capsaicin Application

The insular cortex is composed of functionally diverse subregions, which are involved in gustatory and olfactory processing, somatosensation, interoception, motivation, and the maintenance of homeostasis (Small et al., 1999; Craig, 2002, 2009; Olausson et al., 2002). The involvement of the insular cortex in autonomic functions has been studied extensively (Craig, 2002; Beissner et al., 2013; Cechetto, 2014). Anterior insula and left posterior insula are potentially involved in human autonomic functions (King et al., 1999; Cechetto, 2014). Recently, the autonomic functional organization of the insular cortex has been revealed to be gyri-specific by the three autonomic manipulations: Valsalva maneuver, hand grip challenge, and cold pressor challenge (Macey et al., 2012). In particular, the ASG was found to be involved in sympathetic regulation as assessed by electrodermal activity and high-frequency heart rate variability (Beissner et al., 2013).

Transient increases in heart rate and blood pressure, and tympanic temperature were observed immediately after ingesting, chewing, and spitting out hot red pepper (Hachiya et al., 2007). In the present study, the ASG was more strongly activated by capsaicin compared to the M/PSG (**Figures 6C–E**). The fingertip temperature measured after capsaicin application was significantly higher compared to the control (**Figure 7A**). The bilateral ASG showed a significant positive correlation between fingertip temperatures and BOLD signals (**Figures 7Ba,Ca**). These results suggest that the ASG plays a crucial role in inducing autonomic responses following capsaicin administration, as reflected in fingertip temperature increases. Furthermore, the significant positive correlations between the effect size in the hypothalamus and those in the bilateral ASG (**Figures 8Ea,Fa**) suggest that the ASG activity caused an increase in the fingertip temperature through the activation of the hypothalamus. Indeed, the peak coordinate found as significant in the hypothalamus by the group analysis of the capsaicin responses corresponded to the dorsomedial hypothalamic nucleus, which is known to be involved in the control of body temperature (Nakamura, 2011).

After conjunction analysis between all the responses to the respective taste stimuli, insula, and postcentral gyri were prominently activated following application of tastants on subject’s tongue, and the precentral gyrus was activated during pressing the button. Other regions including bilateral supplementary motor area, bilateral middle cingulate cortex, bilateral middle frontal gyrus, and bilateral cerebellum were also activated (**Table 1**). These regions were also reported to be activated by the nociceptive responses to heat, capsaicin, or mechanical stimulation applied to the hand or forearm skin by using positron emission tomography and fMRI (Peyron et al., 2000). However, the supplementary motor area and cerebellum were known to be involved in the regulation of sympathetic activity (Beissner et al., 2013), as revealed by significant correlations between fMRI signal and instantaneous high frequency power of heart rate changes (Napadow et al., 2008).

### Do Capsaicin-Induced fMRI Responses in the Insular Cortex Represent Pain Perception?

In the present study, capsaicin was applied at a concentration of 65 μM, which is 10–30 times lower than that contained in tabasco sauce and similar to that of curry sauce. In a previous study, capsaicin was applied at 44 μM which caused neither pain sensation nor activation of the ASG (Rudenga et al., 2010).

Robust activations in the anterior and posterior long gyri (A/PLG) of the insular cortex during nociceptive stimulation were consistently shown in fMRI studies (Apkarian et al., 2005; Duerden and Albanese, 2013). These posterior parts of the insular cortex together with inner opercular cortices form a first-order nociceptive matrix, and a second-order perceptual matrix is composed of the middle and anterior insular cortices, the anterior cingulate gyrus, anterior frontal, and posterior parietal areas (Garcia-Larrea and Peyron, 2013). It was also reported that nociceptive input was first processed in the posterior insula and then conveyed to the anterior insula using Stereo-Electro-Encephalography before neurosurgery (Frot et al., 2014).

In the present study, a group analysis of the comparison [2 × Capsaicin–NaCl–AS] revealed that capsaicin activated the ASG more potentially than the M/PSG without significant activation of the A/PLG (**Figure 5** and **Table 4**). As the A/PLG is the first-order nociceptive matrix, these observations suggest that capsaicin did not cause pain sensation but activated parasympathetic nervous system to cause an increase in the fingertip temperature. Usually, in response to cold acclimation of the fingertip, the contraction of fingertip endartery would be caused by α2 adrenergic action to prevent the temperature loss (Nakamura, 2011). However, under the resting condition with oral administration of capsaicin, such adrenergic response would not occur whereas adrenergic action on the heart induced by capsaicin would increase blood flow in the fingertip endartery to increase the fingertip temperature.

## Author Contributions

YK and NS designed research; SK and HS performed research; AS, HT, and YY acquired fMRI data; SK, HS, MS, and HT analyzed data; SK, HS, YK, and NS wrote the paper.

## Conflict of Interest Statement

The authors declare that the research was conducted in the absence of any commercial or financial relationships that could be construed as a potential conflict of interest.
